# The necessity of examining patients’ social behavior and teaching behavior change theories: curricular innovations induced by the COVID-19 pandemic

**DOI:** 10.1186/s12909-021-02582-2

**Published:** 2021-03-08

**Authors:** Danette Waller McKinley, Saeideh Ghaffarifar

**Affiliations:** 1 Foundation for Advancement of International Medical Education and Research (FAIMER), Philadelphia, USA; 2 Medical Education Research Center, Health Management and Safety Promotion Research Institute, Tabriz University of Medical Sciences, Tabriz, Iran

**Keywords:** Social behavior history, Behavior change theories, Medical curriculum, Curricular innovation

## Abstract

During the COVID-19 pandemic, despite many widespread calls for social distancing, recommendations have not been followed by some people and the high rate of non-compliance has significantly affected lives all around the world. It seems that the rate of non-compliance with the recommendations among medical students has been as high as the rest of the other youth. In the time that students are removed from clinical environments and most physician teachers are strained in providing services to patients, medical students can be trained in interdisciplinary behavior change counseling programs and they can be employed in delivering virtual consultations to the patients referred to medical centers.

In this quick review, we provide an argument regarding the importance of integrating the topic of patients’ social history into the undergraduate medical curriculum and the necessity of teaching theories of behavior change to medical students. Hypotheses are proposed that focus on the importance of integrating behavioral and social sciences into the medical curriculum and to teach theories or models of behavior change to students. Health professions educators can design and implement interventions to teach hypothesized models of behavioral change to medical students and evaluate the effectiveness of those interventions. The impacts of such educational interventions on increasing people’s compliance with recommendations to improve public health can be evaluated as well.

**Supplementary Information:**

The online version contains supplementary material available at 10.1186/s12909-021-02582-2.

## Background

For many years, training medical students to be competent in the cultural and social aspects of health care has been emphasized by scholars and institutions around the world. Integrating behavioral and social sciences (BSS) into the American undergraduate medical curriculum was recommended by the Institute of Medicine (IOM) in 2004 [1] and guiding the “strategic development of an educational institution” by “social, economic, cultural and environmental determinants of health” was recommended by Boelen and Woolard in 2009 [2].

In the last decade, different strategies and methods have been employed to teach BSS to medical students. Medical students are involved in behavior counseling with patients. Considering the areas featured by Healthy People 2030 [3], social determinants of health (SDoH) are situated among other topics into the undergraduate medical curriculum in some universities. In order to reduce the rate of premature deaths and increase the human beings’ quality of life, medical students are educated to pay special attention to the role of social factors affecting health [4]. Many single or multi- institutional interventions, with different degrees of impact, are implemented to train medical students to advice and advocate behavior change in patients.

Despite much invaluable advancement in medical education, concerns have been expressed that medical students are not adequately prepared to be competent in behavior-change counseling. A systematic review on “behavior change counseling curricula for medical trainees” published in 2012 reported that student satisfaction and/or knowledge, not their performance (behavior) are considered as outcome measures in most studies [5]. It can be inferred from the findings of this review that present medical curricula lead to only an increase in learners’ knowledge and satisfaction and may not be able to change the actual performance of students.

The shortcomings of the present curricula in changing the actual performance of medical students may be exacerbated during the COVID-19 pandemic. Studies indicate that despite many widespread calls for social distancing during this recent pandemic, many regulations and recommendations have not been followed by some people in the world. Such non-compliance has affected the quality of life of many people around the world. The rate of premature deaths among the population has unfortunately increased. In the U.S, 39.8% of participants of an online survey were non-compliant. Eighteen- to thirty-one-year-olds were less likely to follow orders of social distancing (compliance rate = 52.4%) [6]. Given many concerns in the time of COVID-19, most medical students have been removed from clinical environments and they are not directly involved in COVID patients’ care. The pandemic provides an opportunity to advance medical education through “active curricular innovations” [7]. Contemplating the shortcomings of the present curricula in teaching the role of BSS in changing health-related behaviors, revision of the undergraduate medical education curriculum, with a special focus on patients’ social behavior history, seems necessary.

In 1951, the role BSS can play in medical education was elaborated. Later, opportunities to enhance BSS training in medicine were provided by many universities in the US. The first behavioral science department in a medical school was established in the University of Kentucky in 1959. Since then, repeated efforts have been made to integrate the social sciences into medicine, but several barriers have persisted [8]. That is why, in 2004, IOM recommended including BSS in medical curricula, in six different domains. These domains include health policy and economics; social and cultural issues in health care; mind-body interactions in health and disease; physician role and behavior; patient-physician interaction and patient behavior [1].

Focusing specifically on patient behavior, as one of the six BSS domains recommended by IOM, this review is intended to look at the extent to which examining patients’ social behavior and teaching behavior change theories are included in undergraduate medical curriculum.

## Methods

To conduct this quick review, bibliographic databases including MEDLINE (via Ovid), PubMed, ERIC (via EBSCO) and Cochrane Library (via Ovid) were searched. Using keywords: “medical education”, “medical curriculum”, “medical training”; “social science”, “behavioral science”, “social behavior”, articles published through June 2020 were searched. A combination of controlled vocabulary, free text keywords and their synonyms were used in search strategy. The search results were limited to the publications after 2000 and those which were focused on undergraduate medical education. Our search strategy of Ovid MEDLINE(R) is provided in Additional file 1. One thousand five records were identified through database searching. Duplicates were removed and 886 records were screened. Fifty-nine full texts were reviewed for eligibility. Records, which were not about BSS integration into undergraduate medical education and those which were not published in English were excluded.10 articles were found based on ancestry searching and forward tracing as well. In all, 21 studies relevant to BSS instruction for medical students were reviewed. The characteristics of the included studies are presented in Table 1.

**Table 1:** Characteristics of the included studies (*n* = 21) until June 2020

Study characteristics		Reference numbers of the included studies
**Type of study**	Editorial	19
	Editorial review	31
	Brief review	2,10,15
	Systematic review	5,30
	Original research	8,9,11,12,13,17,18,20,29,32,33,34
	Others	4,16
Type of information	The importance and necessity of BSS integration	2,4,5,9,11,13,15,16,24
	Barriers to BSS integration	8,29,30
	The approach to BSS integration	8,12,17,18,29,30,31,32
	Learning context	8,9,29
	The content required to be covered in curriculum	8,9,17,29,30,31
	Teaching method of BSS	10,11,15,17,21,31,32
	Assessment method of BSS integration	11,17,18,19,20,30,31
	Time of BSS integration	8,10,11,18,19,30,31
	Hours required to teach BSS	18,31
	Teachers’ characteristics	11,29,30,31,34
	Departments required to be involved	9,12,25,31,33
Country	United states	4,5,8,9,10,11,15,16,17,18,19,33,34
	United kingdom	13,20,29
	Canada	2,19,32
	Iran	12,30
	France	2
	Germany	31
	Australia	31

## Results

The characteristics of the included studies are presented in Table 1.

The reviewed studies were summarized to elaborate the importance of examining patients’ social behavior and the required changes in the curriculum; and to identify lessons learned.

### The importance of examining patients’ social behavior

About half of all reasons for mortality and morbidity in the United States are correlated with social and behavioral factors [9]. Behavioral medicine has been introduced as “some of medicine’s most powerful tools for reversing dangerous health trends in the U.S.” [10]. It seems that synthesizing the micro biomedical perspectives with macro public health ones is an urgent need to provide optimal health for patients. That is why it is believed that “all health is global health” and “all medicine is social medicine” [11].

In a content analysis study, in which medical faculty members’ perspectives on the necessity of incorporating BSS into the medical curriculum were explored, six themes were emerged. The results of this study showed that integrating BSS into medical curriculum leads to facilitating health behavior changes [12].

Routinely, students in most medical schools do not explore their patients’ social behavior history. In order to deliver quality care, they are encouraged to gather data by conducting a history interview of patients’ problems in full. In practice, however, they just do their best to ask enough questions to document their patients’ past and present history of illness, as well as family and drug histories. Indeed, taking and analyzing the history of patients’ social behavior usually plays a small role in medical students’ diagnostic and therapeutic reasoning. For instance, participating students in Peterson and colleagues’ study were exposed to a reformed curriculum at Oregon Health and Science University and were educated about BSS during their first 2 years at medical school. They reported that they were not able to internalize and use BSS values in the second half of their medical training. Indeed, despite being taught about approaches to guide patients’ behavior, students were unable to translate the training received into their practice [8].

The COVID-19 pandemic highlighted the necessity of integrating the topic of taking and analyzing patients’ social history into the undergraduate medical curriculum. A curricular change focused on taking and analyzing patients’ social behavior as a crucial competency along with more opportunities to practice and rehearse this competency can be provided to medical students. The more they practice, the more they recognize the role of the patients’ social behaviors in improving health outcomes. Such training has been shown to improve students’ understanding and attitudes towards patients with chronic disease [13]. By so doing, the likelihood of change in medical students’ attitude towards the role of social behaviors in ensuring health of communities will increase and they could become more motivated to adopt preventive behaviors in counseling their patients. Most importantly, students who have received the necessary training in analyzing patients’ social behaviors can be employed in implementation of health education programs in the time of pandemics. So, while volunteering in many health education initiatives, medical students can participate in caring for patients through tele-health environments [14].

### Required changes in the curriculum

In all, reviewing the 21 published studies up to June 2020 revealed the fact that in recent decades, “the line between public health and medicine has blurred” [11]. Behavioral medicine, after surgery and pharmacology, has been the third major revolution in improving human health; however, highly effective behavioral treatments are under -recognized or rejected. BSS is poorly appreciated in medical curricula [15]. The biomedical mindset of physicians has not been fully compatible with BSS; and BSS education has had its own problems globally.

Although the Liaison Committee on Medical Education (LCME) and other accreditation bodies in the world have emphasized the need to consider BSS in accreditation of medical schools, incorporation of BSS in the medical curriculum has been variable at many schools around the world [12] and social medicine is taught at less than 10 % of medical schools that are accredited by the LCME [11].

It is stated that BSS education should be multidisciplinary and requires systematic attention to cover BSS in the curriculum in order to increase students’ ability to diagnose patients in a broad social context [12]. Most publications have reported the importance and necessity of integrating BSS into the medical curriculum, and there are very few studies in which BSS integration has been described based on the different components of the curriculum (including teachers’ characteristics; appropriate educational context and BSS teaching and assessment methods) [12].

There is no agreement on when to integrate BSS into the medical curriculum and the content that needs to be integrated [10]. What is clear is that teaching BSS to medical students improves their ability to appreciate the impact of behavior on patients’ wellness and health [16] and to detect the possible reasons of patients’ suboptimal health outcomes [11].

Given the ever-increasing burden of medical schools, static number of teaching hours, explosive production of the content and incredible growth of disciplines, it seems that proper content for integration should be identified through a valid and reliable process [9]. It is recommended that every decision, about the topics, teaching and assessment methods, funding, valuing and management methods of teaching material should be established in “the context of competing disciplines” [9].

Improving the educational context as much as choosing the most appropriate content for integration can be crucial in increasing the clinical application of knowledge by students. For this reason, “no content without context” has been considered as the basis for integrating BSS into the pre-clerkship curriculum at University of California Los Angeles (UCLA). The instructional planning at UCLA has been performed by collaboration of students. Their innovations in repeating the intended content in interdisciplinary blocks, in flipping the courses, in creating cross-disciplinary case vignettes, in incorporating innovated Problem Based Learning (PBL) strategies, and in integrative summative assessments has prepared students to enter clinical wards with a systematic approach for discovering patients’ problems [17].

At University of California San Francisco, “need -to- know” content was identified through a multi-model procedure and BSS content was addressed in six different domains through a four-year integrated curriculum. The domains included “mind–body interactions; patient behavior; the doctor’s role and behavior; doctor–patient interactions; social and cultural issues in health care, and health policy and economics” [9].

The protocols written for training BSS cannot be taught by lecturing alone [15]. The content of BSS has been innovatively integrated into the preclinical medical curriculum across 77 cases in a PBL curriculum, at UCLA during a seven-week period [10].

BSS at Southern Illinois University is taught for first-year medical students in 11.5 weeks. Initiating each session with a clinical case, mentorship activities, “tutor-group and resource sessions”, recording the learning issues into an electronic filing system, performance-based midterm and summative examinations based on the compromised learning issues and remediation units are among the initiatives to create a positive learning experience in their hybrid curriculum [18].

After a reform with multidisciplinary approach in 2007 at Harvard Medical School (HMS), first year medical students are required to attend an integrated “Social Medicine and Global Health (ISM)” course. During the course, they learn to provide solutions to the clinical cases, which are developed by a team of physicians and other health care professionals. Along with ISM, students make home visits and write reflective assignments. Hidden curriculum has been highly considered in designing this integrated curriculum at HMS. In Harvard’s reform, in which the BSS has been integrated to preclinical medical curriculum, provision of interested faculty members with “curriculum time and support” have been considered essential to develop such invaluable courses” [11].

Teaching BSS alone does not warrant application of behavior change theories by medical students to visit patients. Standardized and valid evaluation systems and tools are required to ensure applying BSS and retaining humanistic attitudes by students. Since April 2015, assessment of BSS has been included in the third part of the Medical College Admission Test (MCAT) in order to evaluate candidates’ understanding of the influence of behavior on wellbeing and health; and to enhance the medical culture as a bio-psychosocial enterprise [19].

Assessments are better to focus on clinical applications of BSS to stimulate students’ deep learning [17]. So, to properly assess the knowledge of BSS, Multiple Choice Questions (MCQs) and Short Answer Questions (SAQs) alone are not enough [20]. Newer assessment methods need to be investigated to evaluate students’ deep learning and applied knowledge. At Lancaster Medical School in the United Kingdom, students’ applied knowledge of BSS is being evaluated by Scenario-based assessment, which has been aligned with PBL curriculum [20].

As it is inferred from international scholars’ experiences and overviews, it is recommended to start BSS training from first years of studying at a medical school (preclinical years) [10, 11, 19]. Longitudinal integration of BSS into the medical curriculum and interweaving BSS to a “case-based, clinically oriented medical curriculum” is recommended as well [12, 18]. To provide COVID-19 behavior change theories-oriented specific training, Massive Open Online Courses (MOOCs) can be designed [21] and offered to medical students, as electives, on every available online learning management system. Courses can be flipped and students’ online individual learning activities can be joined with online virtual classes. MD-PhD teachers can facilitate students’ case-based discussions and supervise students’ online activities and provide them with constructive feedback. The engaged students can be provided with the feedback from their fellow learners as well.

According to the IOM call, BSS integration should address six different domains including social and cultural issues in health care, patient-physician interaction and patient behavior [1]. Students should be taught about communicating with specific vulnerable populations such as people with low health literacy. Medical students should be instructed to avoid using medical terms to interview patients with low health literacy. Instead, they should be able to use some plain alternatives. They should focus on only a few main key messages. They should speak slowly. Using a teach-back method, they should ask their patients to repeat their instructions back to them in their own words. They can also develop some written materials, with an easy-to-understand and picture-based format [22] and send them to their patients via social networks. In case of a language barrier, employing interpreter services, which are provided by tele –communicators, recruiting multilingual students and non-verbal communication [23] will implicitly improve the physician-patient relationships and quality of care.

### The importance of including behavior change theories in undergraduate medical curriculum

For medical students to be able to have a comprehensive interview with patients, they need to have previously been taught behavior change theories. Because, teaching the principles of interviewing patients without having a basic knowledge of behavior change theories is similar to teaching gene therapy to learners who have not previously learned the basics of genetics [16].

In order to involve students in behavior change counseling programs and to employ them in delivering virtual consultations, it is necessary to deepen their knowledge of the factors which predict patients’ social behavior and train them how to obtain patients’ social behavior history. In this regard, serious curricular development in terms of teaching behavior change theories to medical students is an imperative requirement. Expanding medical trainees’ knowledge about theories of behavior change and empowering them to make good use of theories in predicting patients’ non-compliant behaviors will increase the effectiveness of behavior change consultations [24].

Medical students, who have previously been taught behavior change theories, will be able to have a comprehensive interview with COVID patients. For instance, when an individual does not perceive him/herself susceptible to the COVID-19 and its harmful consequences, a well-trained, knowledgeable student will scrutinize his/her preventive behaviors by specific questions based on the various constructs of behavior change theories, including HBM. By doing so, he/she will realize the low perceived susceptibility of that person about COVID and will consequently provide tailored and specific education for that person to adopt preventive health behaviors. Indeed, identifying the determinants of each individual’s behavior to adopt preventive behaviors requires a great deal of time and thorough understanding of behavior change theories. During the pandemics such as that of COVID 19, when teaching physicians are at the forefront of disease control, medical students in their preclinical years have to spend most of their time at home. These times are great opportunities to ask medical students to make online calls with patients and their families in order to sharpen their consultation skills by managing comprehensive interviews with patients. That is why it is believed that during the COVID-19 pandemic, behavior change consultations by students who have mastered the theories of behavior change can decrease the rate of non-compliance with COVID-19 social distancing and save the lives of many people, including health care providers. By so doing, the burden of diagnosis and treatment in the health care systems will be decreased. “In the U.S., where the crisis is expected to cost nearly 3% of its GDP” [25], the economic rippled effects of the COVID-19, which have profoundly affected governmental expenditures for health purposes, will be neutralized.

There are many other theories and models, developed for health-related behaviors, which students need to be familiar with in order to explain and predict people’s health behaviors. According to the systematic review on behavior change counseling [5], the Trans Theoretical Model (TTM) is the most common model of behavior change, which is taught in behavior change counseling programs in medical schools. The TTM, like many other behavior change theories and models, has its own limitations and cannot explain all health behaviors alone. The Health Belief Model (HBM) [26], the Theory of Planned Behavior (TPB) [27] and Social Cognitive Theory (SCT) [28] are those that address changes in behavior at the individual level.

Explaining and analyzing the high rate of non-compliance with social distancing in the time of the COVID-19 can help medical students learn the constructs of the HBM and plan for disease prevention in society. Studying the constructs of TPB, medical students learn that intention is the most important predictor of people’s behavior. They also learn that in order to change people’s intention about a behavior, such as social distancing, they should consult people to change their attitude toward that behavior, their subjective norms and their perceived behavioral control [26]. Mastering SCT, medical students will be able to analyze personal, behavioral and environmental determinants of people’s thoughts and actions. In this regard, educational researchers can employ SCT to analyze the role of social media in “informing, enabling, motivating, and guiding” people to comply social distancing recommendations in the time of COVID-19 [27].

Using various constructs of behavior change theories or models, medical students can analyze the high rate of non-compliance with recommendations during the COVID-19 pandemic. Some hypotheses, which may explain the high rate of non-compliance with recommendations during the COVID-19 pandemic, are presented in Table 2. These hypotheses have been proposed merely to emphasize the importance of integrating behavioral and social sciences into the medical curriculum and to teach theories and models of behavior change to students. These are some examples based on some constructs of the HBM, TPB and SCT. Undoubtedly, the list of these hypotheses can be further elaborated based on the other constructs of HBM, TPB and SCT, or the constructs of the other theories and models that were not mentioned in this paper.

**Table 2:** Possible explanations of the high rate of non-compliance with recommendations during the COVID-19 pandemic

Theory or model	Construct	Hypothesis^a^
HBM	Perceived susceptibility	People do not perceive themselves susceptible to the COVID-19 and its harmful consequences.
HBM	Perceived severity	People do not believe in the severity of the condition or the extent of harm form the COVID-19.
HBM	Cues to action	People are not faced with proper triggers (whether internal or external) to accept the recommendations about the COVID-19.
TPB	Behavioral intention	People have not seriously considered the COVID-19 yet and do not have a serious plan to follow the recommendations.
TPB	Attitude towards the behavior	People do not appreciate the value of following the recommendations about the COVID-19.
TPB	Subjective norm	People believe that the significant others in their lives will not support their performance to comply with the COVID-19 recommendations.
TPB	Outcome evaluations	People underestimate the value of the consequences of following the recommendations with the COVID-19.
SCT	Outcome expectancies	
SCT	Self-efficacy in overcoming impediments	People are not confident enough that they can overcome barriers when following the recommendations with the COVID-19.
SCT	Knowledge	People have not learnt adequate facts and have not gained enough insights about following the recommendations with the COVID-19.

^a^ Proposed based on constructs of behavior change theories or models

### The lessons from previous efforts and tips for curricular innovations

Before any planning to highlight the patients’ social behavior history in medical education, reviewing the barriers to the integration of the social sciences into the medical curriculum can be helpful. In a survey, educators from all medical schools in the UK reported on the barriers they experienced in teaching the social sciences. They reported that there is often a lack of qualified staff to teach and translate BSS to medics. Many senior educators are not interested and committed to integrate BSS in medical curricula. They do not encourage the essential culture modification. Competent social educators are not given priority in faculty recruitments. Medical training is predominantly compatible with a biomedical mindset. Medical curricula and timetable are usually crowded and there is not enough space for formal integration of BSS. Assessments do not fit with students’ performance and do not drive their true learning [29].

Barriers to integration of BSS in the undergraduate medical curriculum are explored in a systematic review in 2016 as well [30]. It is interesting that all these barriers have remained similar during the decades. Although strategies to overcome these barriers, which are recommended in this review and other studies, have focused on BSS domains other than patient behavior, reviewing those strategies will help in planning for any curricular innovation in terms of teaching behavior change theories in undergraduate medical curriculum and examining patients’ social behavior.

BSS can be effectively integrated in undergraduate medical curricula if BSS in undergraduate medical curriculum moves from “nice to know” to “need to know” [29, 31], and the approach of medical schools in teaching BSS changes from a luxury and trial and error state to formal and continuous training [31]. By so doing, integration should be considered as a key to teach BSS within an already crowded curriculum [29] and theoretical frameworks should be provided for “meaningful integration of extended knowledge domains into curriculum” [32].

Basic science teaching should be extended and supported with BSS too [32].

To integrate BSS in undergraduate medical curricula, teaching BSS should not be limited to the first half of studying at medical schools. It should be integrated throughout the years of study at medical schools. Supplements should also be offered later in clinical years [8, 30, 31]. BSS should be consistently included in both clinical and basic courses [30]. Moreover, adequately experienced and qualified BSS teachers should be employed and involved in teaching BSS at medical schools [29, 31], and BSS- specialized departments should be established within medical schools. In this way, cooperation between BSS specialists and medical doctors will reduce disagreement between medical doctors and BSS professionals [25].

To deliver effective BSS integration and training, medical teachers should be sensitized with proper knowledge and competencies to teach BSS. In addition, faculty development training courses for clinician teachers are recommended so that teachers employ BSS in their daily training [31]. It is also recommended that BSS courses are supervised by BSS specialists [30]. Furthermore, BSS training should be dynamically delivered by mutual collaboration of clinical, biomedical science and behavioral science departments [33]. Training MD-PhDs, with combined knowledge and experience in both medicine and health education, can significantly increase social accountability of medical schools in this regard [34].

BSS integration and training will be done effectively if attending physicians, residents and senior students are role models for employing BSS in clinical settings [8], and if a complete list of learning objectives are decided in order to acquire clinical skills related to BSS [30, 31]. It is also recommended that innovative methods to teach BSS and stimulate students’ learning should be developed [30, 31] so that students can transfer the new content in learning and transfer the learnings out to the new ones [32].

Based on the existing literature, delivering BSS by just “discrete lectures” or “stand-alone additional sessions” should be avoided [32]. Educational materials and modules should be developed and made available to all students so that students can use them in a completely flexible manner as needed [31] and the content of BSS should be reflected in student assessment and certifying examinations [30, 31].

In order to accelerate the progression of integration of BSS into medical curricula, working parties are better to be formed from different school, university or country levels [31]. Of course, prior to any planning to improve integration of BSS in the medical curriculum, hidden curricula at medical schools should be scrutinized in order to explore “institutional policies, evaluation activities, resource-allocation decisions and institutional slang” [29, 31]. BSS content should also be translated to practical points so that the content can be tied to in learners and teachers daily practice [8, 29, 30].

When integrating BSS in curricula, it is important to note that the culture change at medical schools is better to be promoted by senior teachers [29]. Students’ early exposure to community and clinical settings best planned so that they realize and appreciate the applicability of BSS in biomedicine [8, 29]. During such early exposures, medical students should be provided with opportunities to gain rewarding insight to patients’ social behavior so that their thinking can be strengthened in early years of studying at medical school [33].

To increase the impact of integration, it is important to note that formal and regular feedback from students who have been trained with reformed curricula should be taken [31], and incentive policies, such as rewards/awards for effective projects or strategies of integrating BSS into medical curricula should be applied [30]. In this regard, sufficient fiscal support should be available to move BSS training into “the mainstream of medical education” [30].

In order to enhance students’ motivation to learn and practice BSS, competitive environments should be developed [30], and teaching BSS content should be prioritized in terms of students and social needs [30].

## Conclusions

Educators and researchers can design and implement interventions based on teaching behavioral change theories or models to medical students and evaluate the effectiveness of those interventions in changing medical students’ practice, their patients’ health behavior and health outcomes in society. The impacts of such educational interventions on increasing people’s compliance with behavioral changes such as social distancing recommendations can be evaluated as well.

## Supplementary Information

**Additional file 1.**

## Data Availability

No applicable.

## Abbreviations

GDP: Gross Domestic Product
HBM: Health Belief Model
HMS: Harvard Medical School
LCME: Liaison Committee on Medical Education
MCAT: Medical College Admission Test
MCQ: Multiple Choice Question
PBL: Problem Based Learning
SAQ: Short Answer Question
SCT: Social Cognitive Theory
SDoH: Social Determinants of Health
TPB: Theory of Planned Behavior
TTM: Trans Theoretical Model
UCLA: University of California Los Angeles
