# Supplementary material for: The necessity of examining patients’ social behavior and teaching behavior change theories: curricular innovations induced by the COVID-19 pandemic
Source: BMC Med Educ. 2021 Mar 8;21:150. doi: 10.1186/s12909-021-02582-2 (PMC7938272; doi:10.1186/s12909-021-02582-2)
Supplement: Supplementary file 1 — Additional file 1. [file 12909_2021_2582_MOESM1_ESM.docx]

Database: Ovid MEDLINE(R) and Epub Ahead of Print, In-Process & Other Non-Indexed Citations, Daily and Versions(R) <1946 to June 12, 2020>

Search Strategy:

--------------------------------------------------------------------------------

1 ((medical or clinical) adj3 curricul$).hw,kf,tw.

2 ((medical or clinical) adj3 train$).hw,kf,tw.

3 ((medical or clinical) adj3 educat$).hw,kf,tw.

4 1 or 2 or 3

5 ((behavior* or behaviour* or social) adj4 science$).hw,kf,tw.

6 (social behavior* or social behaviour*).hw,kf,tw.

7 (patient* adj3 communication*).hw,kf,tw.

8 5 or 6 or 7

9 4 and 8

10 (undergraduat* or under-graduat*).hw,kf,tw.

11 9 and 10

12 limit 11 to yr="2000 -Current"

***************************
